# A fungi hotspot deep in the ocean: explaining the presence of *Gjaerumia minor* in equatorial Pacific bathypelagic waters

**DOI:** 10.1038/s41598-024-61422-7

**Published:** 2024-05-08

**Authors:** Massimo C. Pernice, Irene Forn, Ramiro Logares, Ramon Massana

**Affiliations:** Departament de Biologia Marina I Oceanografia, Institut de Ciències del Mar-CSIC, Barcelona, Spain

**Keywords:** Microbial ecology, Fungal ecology, Water microbiology

## Abstract

A plant parasite associated with the white haze disease in apples, the Basidiomycota *Gjaerumia minor,* has been found in most samples of the global bathypelagic ocean. An analysis of environmental 18S rDNA sequences on 12 vertical profiles of the Malaspina 2010 expedition shows that the relative abundance of this cultured species increases with depth while its distribution is remarkably different between the deep waters of the Pacific and Atlantic oceans, being present in higher concentrations in the former. This is evident from sequence analysis and a microscopic survey with a species-specific newly designed TSA-FISH probe. Several hints point to the hypothesis that *G. minor* is transported to the deep ocean attached to particles, and the absence of *G. minor* in bathypelagic Atlantic waters could then be explained by the absence of this organism in surface waters of the equatorial Atlantic. The good correlation of *G. minor* biomass with Apparent Oxygen Utilization, recalcitrant carbon and free-living prokaryotic biomass in South Pacific waters, together with the identification of the observed cells as yeasts and not as resting spores (teliospores), point to the possibility that once arrived at deep layers this species keeps on growing and thriving.

## Introduction

The bathypelagic ocean is one of the largest reservoirs of carbon on the planet, both particulate and dissolved. The bioavailable part of this deep carbon pool, also known as labile carbon, decreases along the conveyor belt, following the aging of the water masses, being higher in the Atlantic Ocean compared to the Pacific Ocean^1^. Despite this general decrease, fast-sinking episodes, such as the collapse of a bloom, could inject fresh organic carbon into the deep sea^2^. The remaining part, known as refractory carbon, has been operationally defined as the organic carbon that cannot be degraded at in situ conditions^3^ and increases towards Pacific waters. This increase of refractory carbon along the conveyor belt is mainly explained by the fact that microbial communities act on the total bathypelagic carbon pool reshaping its composition. Among these active microbes, fungi have been proposed to play an important role as decomposers of organic matter, both labile and refractory^4^, with a specialized skillset for the second pool. Despite their importance for the microbial carbon pump, the study of bathypelagic fungal communities and marine fungi in general has long been overlooked.

Pelagic fungi, which are consistently detected in seawater samples, belong mainly to two taxonomic divisions, Ascomycota and Basidiomycota^5^. Species affiliated with these groups, including *Gjaerumia minor*, survive thanks to two main trophic strategies: saprotrophy and parasitism. Saprotrophic fungi degrade and recycle organic matter with extracellular enzymes^6^. In particular, it has been reported that the abundance of fungal CAZymes (Carbohydrate Active enZymes) increases toward mesopelagic waters^7^, pointing to the central role of fungal catabolism in aphotic environments. A similar pattern was also detected for fungal genes related to protein degradation^8^. The utilization of extracellular enzymes is a more effective digestion strategy if associated with a particulate-attached lifestyle. In bathypelagic waters fungi are important colonizers of these particles, exceeding in some cases the biomass of bacteria^9^. As parasites, pelagic fungi are associated with different hosts, including animals, macroalgae, and microplankton^10–12^. Several environmental variables have been proposed to shape fungal abundance in photic waters, including temperature, particulate organic matter^13^, salinity, depth, oxygen and nitrate^14,15^, whereas less information is available about the drivers of their distribution in the bathypelagic realm. It has been suggested that fungal taxonomic diversity increases with depth, mirroring what happens with prokaryotes^5^.

During the Malaspina 2010 expedition, Basidiomycota was found to be one of the most important microbial eukaryotic groups in the bathypelagic ocean^16^. Their relative abundance, based on 454-pyrosequencing, peaked at equatorial Pacific waters, being the most abundant 18S rDNA sequence identical to *Gjaerumia minor*. This species, previously known as *Tilletiopsis minor,* was recently renamed since the entire taxonomy of *Exobasidiomycetes* has been redefined based on their phylogenetic relations^17^. Although the most frequently reported habitat of *G. minor* is plant material (dead or living), through propagules in the air, this yeast is capable of colonizing other niches^18^, including the deep ocean. This ability to adapt to different environments is well exemplified by the fact that this species has been found to be a pathogen of both plants and humans. *G. minor* is one of the causes of the “white haze” in apple trees^19^, a post-harvest disease that flourishes with humidity, cold (4 °C), and low oxygen conditions^18^, while in the literature, three cases of *G. minor* as a human pathogen in subcutaneous mycosis have been reported^20^, severe pneumonia^21^ and corneal abscess^22^. The ability to adapt to different environments is well-known in fungi, and several yeasts are known to be amphibious^23^. Less information is available about plant pathogenic fungi that cause human infections, although this phenomenon appears to be more common than expected^24–27^. The habitat plasticity of *G. minor,* coupled with its uneven distribution in the bathypelagic ocean, makes this species worthy of deeper analyses.

Here we reanalyzed published sequencing datasets from the Malaspina expedition to report on the distribution of *G. minor* along the vertical profiles of several oceanic stations. We first reprocessed the Illumina sequencing of several vertical profiles^28^ to characterize ASVs (Amplicon Sequence Variants)^29^ and found the ASV-363 which has 100% similarity with *Gjaerumia minor* (NG_063045). In addition, taking advantage of the fact that a strain of *G. minor* is commercially available, we were able to develop and test a new TSA-FISH probe with the aim of confirming the presence of this fungus in marine pelagic samples, describing its morphology, quantifying its abundance and investigating a possible role in the degradation of labile and refractory carbon. Finally, we aim to identify abiotic and biotic parameters that may explain the uneven distribution of this species, with the ultimate goal of better characterizing the trophic dynamics of the bathypelagic ocean.

## Methods

The dataset used here derives from the Malaspina 2010 expedition, an oceanographic cruise that sailed across Atlantic, Indian, and Pacific oceans for 7 months with the main objective of studying the bathypelagic ocean (Fig. S1). Information about the sampling protocols can be found in previously published works: the methodology for bacterial abundance with flow-cytometry and prokaryotic biomass calculation is described in Pernice et al.^30^; DNA extraction, 454-pyrosequencing, and bioinformatic pipeline for deep ocean samples are in Pernice et al.^16^; DNA extraction and Illumina sequencing for 12 vertical profiles (5–4000 m) are in Giner et al.^28^; Illumina sequencing for surface samples of the entire cruise are in Logares et al.^31^; and data for Illumina sequencing in bathypelagic samples are in Junger et al.^32^. Both for the 454 and the Illumina sequencing, the target region was the V4 of the 18S rDNA gene using the primers TAReukFWD1 (5′–CCAGCASCYGCGGTAATTCC–3′) and TAReukREV3 (5′–ACTTTCGTTCTTGATYRA–3′)^33^. All the Illumina raw data (12 vertical profiles, global surface, and global bathypelagic) has been newly analyzed here with DADA2^34^ to define the distribution of the ASV of interest (ASV-363) in the different datasets. Data for fluorescence Dissolved Organic Matter (FDOM) was obtained using parallel factor analysis (PARAFAC) as described in Catalá et al.^35^. Values for FDOM and AOU (Apparent Oxygen Utilization) are from Catalá et al.^36^.

**Figure 1:**
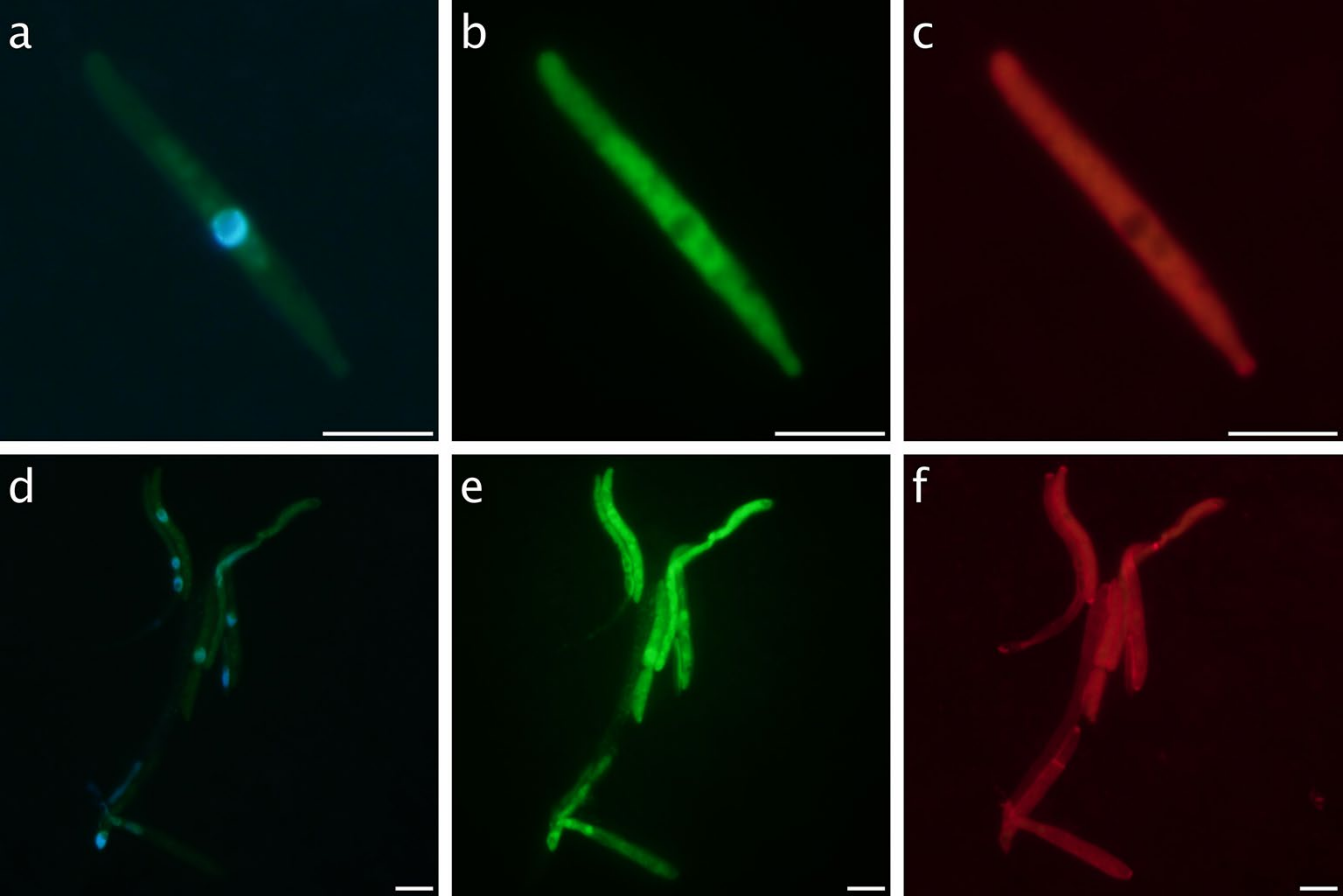
Epifluorescence pictures of cultured cells of *Gjaerumia minor* with triple staining protocols. Panels above show a single elongated cell, possibly a hypha, stained with (**a**) DAPI, (**b**) TSA-FISH (Gmin01 probe conjugated with Alexa-488), and (**c**) WGA conjugated with Alexa-594. The panels below show an entire hyphal structure stained with (**d**) DAPI, (**e**) TSA-FISH, and (**f**) WGA. Nuclei are visible in blue in (**a**) and (**d**), the ribosome-containing cytoplasm in green in (**b**) and (**e**), and the outer chitin cover in red in (**c**) and (**f**). Scale bar is 5 µm.

For TSA-FISH analysis, seawater samples of 475 mL were fixed with 25 mL of 37% formaldehyde (final concentration 1.85%), kept for at least 1 h at 4 °C, and filtered on board on a 0.6 µm pore size polycarbonate filter (25 mm diameter). Filters were stored at − 80 °C until processing. The TSA-FISH probe Gmin01 (5′–CGACCACCATGTGCCCTT–3′) to count *G. minor* cells at the microscope was designed based on the ASV-363 as well as sequences belonging to *G. minor* from NCBI. The probe was checked in silico against the SILVA 138 database and was found to be species-specific: there were no species with 1 or 2 mismatches with the probe, and among the non-targets species with 3 mismatches, there was only one multicellular eukaryote. The optimization of the hybridization conditions was done using a commercial strain of *G. minor* (*Tilletiopsis minor* Nyland fungal strain, JCM No. 8361). A first attempt with the standard TSA-FISH protocol, as described in Pernice et al.^30,37^ adapted from Pernthaler et al.^37^, gave poor results (less than 25% of positive cells in the culture). The addition of helpers to contiguous regions of the probe (HelperA-Gmin1 5′–GCGGGCTCGCGGCGATCAAT–3′; HelperB-Gmin1 5′–ACCAAGTTTGCCCAAGTTTT–3′) increased the results to 65% of positive cells. The use of Wheat Germ Agglutinin conjugated with Alexa Fluor 594 (Thermofisher; WGA 0.01 mg mL^−1^, 30 min at RT), a fluorescent marker staining chitin, confirmed the presence of a thick wall in cells labeled with the probe so it was decided to include an additional permeabilization step. After trying several protocols, the best results were obtained using an incubation for 1 h at 30 °C in a permeabilization buffer (pH 6.5) consisting of 1 × PBS, 1% SDS, 1 mg mL^−1^ chitinase and 6 mg mL^−1^ Glucanex (a cocktail of enzymes isolated from *Trichoderma harzianum* that contains β-glucanase, cellulase, protease and chitinase as in Priest et al.^38^. With this permeabilization step, we reached more than 90% of positive cells (Fig. 1). Finally, the optimal hybridization stringency was determined by keeping the temperature constant at 35 °C and varying formamide concentrations (0–70%) in the hybridization buffer. The optimal value was 20% formamide, the highest concentration before probe signal intensity decreased. We applied the optimized TSA-FISH protocol to a subset of 34 environmental samples from the deepest layer of the Malaspina 2010 expedition.

Microscopic analysis was performed on an Olympus BX61 epifluorescence microscope (Olympus America Inc.) at 1000 × magnification under UV for DAPI, blue light for A488 (TSA-FISH) and green light for WGA. Target cells (two morphotypes, rounded and elongated) were counted by inspecting microscopic transects between 30 and 80 mm (average of 54 mm/sample, equivalent to 540 fields). Pictures were taken on an Olympus DP72 camera connected to the microscope. Biovolume for the rounded morphotype was calculated by assuming a spherical cell based on the average diameter (100 cells measured). The biovolume of the elongated morphotype was calculated assuming a cylinder with 2 half spheres shape^39^ based on the following formula: V = pi * d^2^ * (h/4 + d/6), where h is the largest cell dimension and d is the cross-section of h; average h and d are based on 40 measured cells. We then used the equation of Menden-Deuer and Lessard^40^ to convert cell biovolume to cell biomass: pgC cell^−1^ = 0.216 * (Biovolume^0.939^). Within each sample, average cell biomass times cell abundance counted by TSA-FISH for each morphotype was calculated and then summed to obtain the total biomass of the *G. minor* population.

Statistical analyses (Regression analysis and Pearson correlation) were performed with Rstudio (package Hmisc).

## Results

### Morphotypes

**Figure 2:**
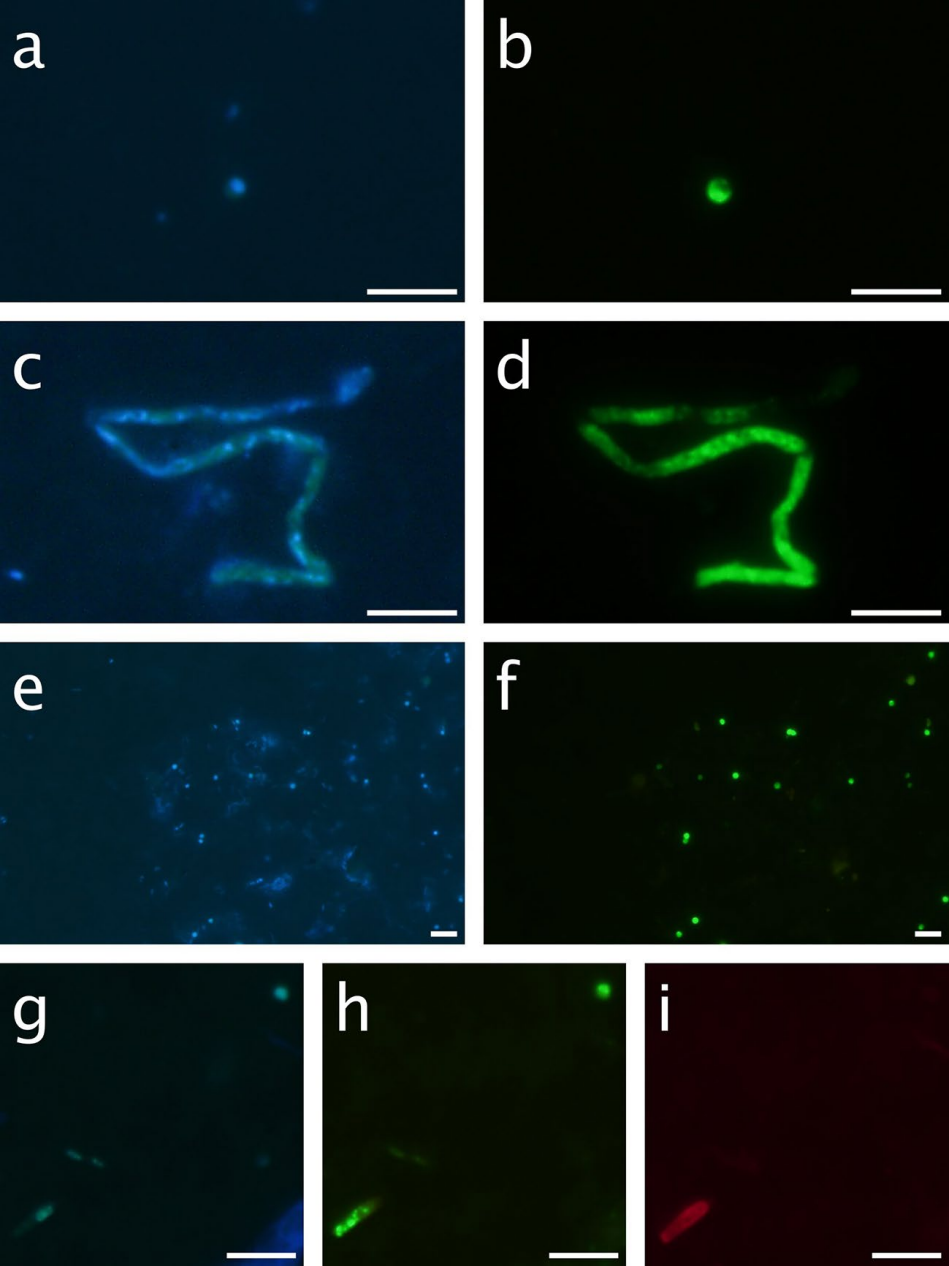
Examples of cells from environmental samples. A rounded morphotype (average size 1.06 µm, probably a yeast) from ST83 stained with DAPI (**a**) and the Gmin01 probe (**b**). A hyphal structure (h = 6.9 µm, d = 1.16 µm) from ST88 stained with DAPI (**c**) and the Gmin01 probe (**d**). A group of rounded morphotype cells embedded in a gel particle from ST97 stained with DAPI (**e**) and the Gmin01 probe (**f**). A field including the two morphotypes from ST92, a hypha in the low-left corner and a rounded cell in the up-right corner stained with DAPI (**g**), the Gmin01 probe (**h**) and WGA (**i**). Note that the chitin staining applies to the hypha but not to the rounded cell. Scale bar is 5 µm.

Two different morphotypes have been identified as *Gjaerumia minor* by the TSA-FISH probe (Fig. 2), one rounded with an average diameter size of 1.5 µm (panels a and b), and one elongated (panels c and d) with an average h (larger cell axis) of 5.2 µm and an average d (smaller cell axis) of 1.5 µm. The rounded morphotype, which represents the majority of cells retrieved, shows a single nucleus per cell, evidenced in the figure by the DAPI signal. This cell type never appears in culture and was not stained by WGA, as it is clearly visible when comparing to the elongated type in Fig. 2g,h and i. So, it was assumed that the rounded morphotype has low chitin content. Although a rounded shape is often associated in congenera species with a teliospore (resting spore, ticker chitin wall, dikarya), our observations (cell size, lack of thick chitin, and the presence of only one nucleus) lead us to consider the rounded morphotype not a resting spore but an active yeast cell. In fact, it has been suggested by the authors that defined the genus *Gjaerumia* that environmental conditions may be responsible for different shapes in unicellular fungi^41^. The elongated morphotype, found only in Pacific waters, is probably a hyphal stage. Sometimes, it is found in chains (Fig. 2c,d) but more often as single cells (Fig. 2g,h and i) with a visible nucleus in the middle as observed also in cultured cells.

### Vertical distribution of the target 18S rDNA sequence

**Figure 3:**
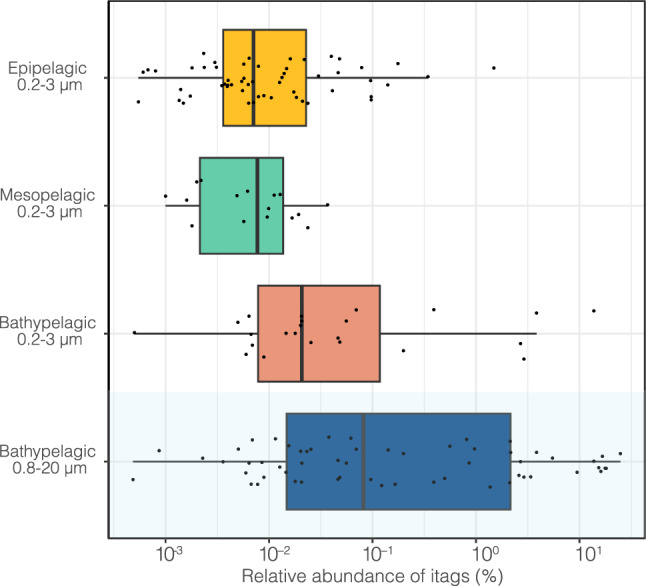
Vertical distribution of *Gjaerumia minor* based on the relative abundance of ASV-363 that is 100% similar to cultured *G. minor* (strain AB7-11). Sequences derive from previously published Illumina datasets^17,25^. Samples from 0.2 to 3 µm size-fraction were grouped in three layers: Epipelagic (3–200 m) including 149 samples, Mesopelagic (200–1000 m) including 32 samples, and Bathypelagic (1000–4000) including 31 samples (zero values are not shown in the graph for better visualization). The last boxplot (in blue) shows the relative abundance in 22 Bathypelagic samples belonging to the 0.8–20 µm fraction (no zero values were found in this dataset).

In order to have a general view of the distribution of *G. minor* in the global ocean, we used already published sequencing data of 18S rDNA genes from the 0.2–3 µm size fraction (Illumina tags grouped in ASVs). The sampling plan was not designed to study fungi and this size fraction likely retrieved all rounded cells but could have missed some elongated ones. The relative abundance of the *G. minor* ASV (ASV-363) was available for 12 vertical profiles from the surface to 4000 m depth and for 124 surface samples of the entire cruise (Fig. 3). The taxonomic identity of this ASV was confirmed by a parallel analysis on deep Malaspina metagenomes^42^ which yielded a *G. minor* MAG (Metagenome Assembled Genome) that featured an ITS rRNA sequence identical to a strain isolated from a pleural effusion of a child with pneumonia (KT149771). ASV-363 tends to increase with depth: the median of the relative abundance was higher in the bathypelagic (0.0146% of tags) than in the epipelagic (0.0000%) and mesopelagic layers (0.0005%). These differences were statistically significant (Kruskall–Wallis test, epi-bathy, *p* = 0.000003; meso-bathy, *p* = 0.001610). ASV-363 was found in 71% of the samples of the bathypelagic realm, while this number was 47% for mesopelagic and 33% for epipelagic samples, further stressing its higher presence in the deeper ocean. Illumina sequencing was also performed in 22 bathypelagic samples from another size fraction (0.8–20 µm). These samples show higher values of the ASV-363 relative abundance, with a median of 0.0829% and a maximum of 24.8499%, being statistically different from the 0.2–3 fraction (*p* = 0.001062). The vertical distribution clearly points to the bathypelagic ocean as the preferred environment for this species, and we decided to focus on the deepest samples for the TSA-FISH analysis.

### Cell abundance in the bathypelagic region and fit with sequencing data

**Figure 4:**
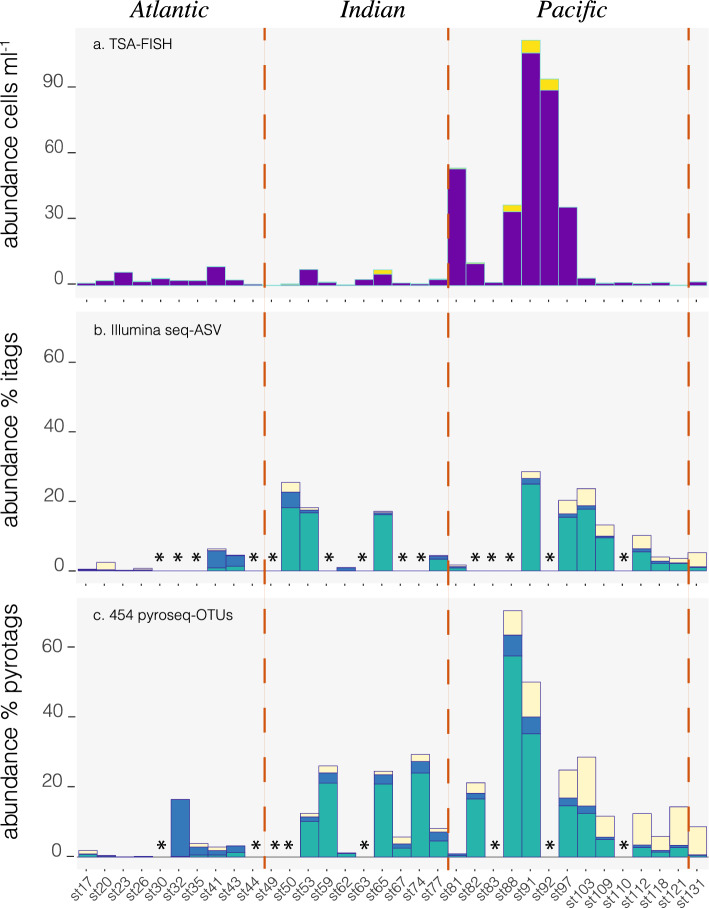
Distribution of *Gjaerumia minor* in bathypelagic waters (deepest sample in each station) along the entire track of Malaspina cruise. Oceanic boundaries are depicted as a red dotted line for the three graphs. (**a**) Cell abundance of rounded cells (probably yeasts, violet) and elongated cells (hyphae, yellow) stained with TSA-FISH in the deepest point of the 34 stations spread across Atlantic, Indian and Pacific Oceans; (**b**) Relative abundance of *G. minor* Illumina tags (ASV_363) in green, of other Basidiomycota tags in blue, and of Ascomycota tags in light yellow; (**c**) Relative abundance of 454-pyrosequenced tags (in 26 of the 34 previous stations) published in Pernice et al.^16^ (size fraction 0.8–20 µm), tags belonging to OTU-6359 (99.7 similar to *G. minor*) are shown in green, other Basidiomycota in blue and Ascomycota in yellow. Illumina and 454 sequencing were done from the same DNA extraction. Asterisks show stations where no sequencing data was available.

TSA-FISH counts targeting *G. minor* were performed on the deepest sample (between 2600 and 4000 m) of 34 stations. Figure 4a shows the cell abundance of the two morphotypes retrieved along the cruise track crossing the Atlantic, Indian, and Pacific Oceans. Cells were observed in all 34 bathypelagic samples inspected, with the rounded morphotype being always more abundant than the elongated one. *Gjaerumia minor* rounded cells had low abundances (less than 10 cells mL^−1^) both in the Atlantic and Indian oceans and were more abundant in Pacific waters, peaking at station 91 with 107 cells mL^−1^. Elongated cells were also more abundant (3–6 cells mL^−1^) where rounded cells were abundant (stations 88-91-92).

A similar pattern of distribution emerged analyzing the relative abundance of *G. minor* 18S DNA tags in Malaspina deepest samples (0.8–20 µm size fraction), after sequencing the same DNA extracts with Illumina (20 samples, Fig. 4b) and with 454-pyroseq (26 samples, Fig. 4c)^16^. As expected, since the two sequencing analyses are based on the same DNA extract, the relative abundance of the specific OTU (by pyrosequencing) and the specific ASV (by Illumina) have a very strong direct relationship (n = 19, R = 0.84, *p* < 0.001). Both datasets found an almost absence of *G. minor* in the Atlantic Ocean, an intermediate presence in the Indian Ocean, and maximum values in the Pacific Ocean. There was a significant moderate direct relationship between the number of rounded cells and the relative abundance of the specific ASV (n = 18, R = 0.54, *p* = 0.02), this correlation is similar considering the specific OTU (n = 26, R = 0.51, *p* = 0.008). These moderate fits can be explained by the typical errors of each approach and the huge difference in filtered volume, which was ~ 120 L for the DNA analysis and only 475 mL for the TSA-FISH. Sequencing also allowed us to put the relative abundance of *G. minor* (in green in Fig. 4b,c) in the context of other fungi, like the rest of Basidiomycota (in blue) and Ascomycota (in light yellow). In the Indian and Pacific Oceans, where *G. minor* is abundant, this single species represents almost the totality (median of 87%) of Basidiomycota. Ascomycota is generally less abundant than Basidiomycota along the cruise track, but follow a similar global distribution, being almost absent in the Atlantic Ocean and abundant in Pacific waters. Ascomycota have an abundance peak at station 103, which is shifted forward along the cruise track with respect to the Basidiomycota peak at station 88.

### Biomass and its relation with AOU and FDOM

**Figure 5:**
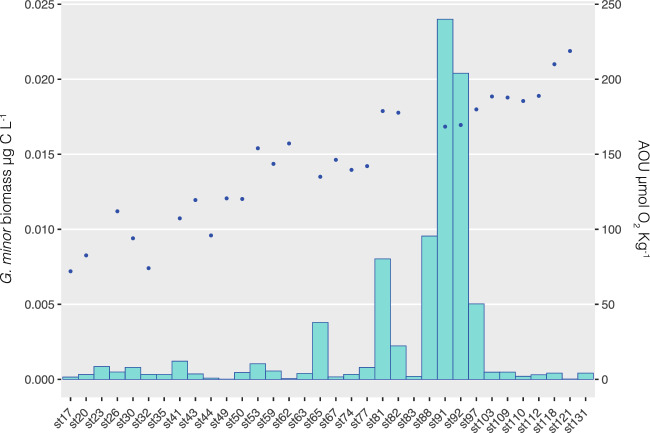
Distribution of *Gjaerumia minor* biomass and Apparent Oxygen Utilization (AOU, µmol O_2_ Kg^−1^) across the Malaspina track. The biomass (expressed as µg C l^−1^) is visualized as a barplot (left y axis) whereas the AOU, corrected by the real value of measured oxygen, is represented by the blue dot (right y axis). AOU is a measure of the amount of oxygen respired in the deep ocean, higher values of AOU are typical of aged waters.

**Figure 6:**
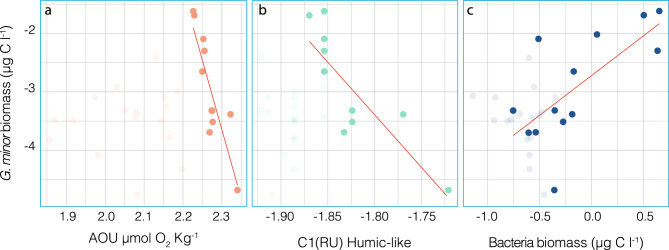
*Gjaerumia minor* biomass relationship with AOU (**a**), C1 component of FDOM (**b**) and bacterial biomass (**c**). Dots belonging to Pacific samples are in bold. Red lines indicate the linear relationships between biomass and FDOM values using only Pacific samples.

The biomass of the two morphotypes combined ranged between 1.74 × 10^−5^ and 2.40 × 10^−2^ µg C L^−1^; globally, the rounded morphotype is the main contributor to the species biomass. The distribution of biomass along the entire cruise is shown in Fig. 5 (bar plot), with the highest values found in equatorial Pacific waters. The points on the graph correspond to Apparent Oxygen Utilization (AOU), which is a proxy for the age of the water mass. Higher AOU values imply older waters. The AOU did not explain the distribution of *G. minor* when the entire cruise track was taken into account. However, when only the Pacific samples were considered (Fig. 6a), there was a strong inverse relationship (n = 11, R =  − 0.88, *p* = 0.0003). Thus, AOU is a good predictor of *G. minor* biomass in Pacific waters (R^2^ = 0.78).

Being AOU strictly connected in this cruise with the carbon composition, in particular with Fluorescent Dissolved Organic Matter (FDOM)^35,36,43^, the relationship between *G. minor* biomass and FDOM was further investigated. The FDOM data for each sample is composed by four peaks, two belonging to humic-like matter (C1 and C2) that are proxies for the refractory carbon, and two associated with protein-like matter (C3 and C4) that represent the labile carbon. Each of these four peaks includes different chemical compounds that are pooled in this analysis as they share the same fluorescence spectra: C1(Ex/Em < 270–370/470 nm, peak A or C), C2 (Ex/Em 320/ 400 nm, peak M), C3 (290/340 nm, peak T), and C4 (270/310 nm, peak B)^44^. C1 seems enriched in terrestrial sources^45^, C2 has been mainly reported in marine samples^44^, whereas C3 and C4 have been attributed to tryptophan and tyrosine^46,47^. We performed a multiple regression analysis with the four peaks for Pacific samples, treating them as independent variables. The entire model explains 75% of the variability of *G. minor* biomass, with the recalcitrant C1 being the only significant predictor (*t*-stat =  − 5.14, *p* = 0.006). Interestingly the same model for bacterial biomass was not significant. The relationship between *G. minor* biomass and C1 is shown in Fig. 6b. Considering only Pacific samples (in bold in Fig. 6b) we found a very strong inverse relationship between *G. minor* biomass and the humic-like C1 peak (n = 11, R =  − 0.84, *p* = 0.011).

Finally, we explored the relationship between *G. minor* biomass and bacterial biomass (Fig. 6c). *G. minor* biomass is extremely low compared with the total prokaryotic biomass, which ranges between 0.12 and 4.49 µg C L^−1^. Nevertheless, the biomass of *G.minor* and prokaryotes are moderately correlated (n = 32, R = 0.55, *p* = 0.0011) and, again, this relationship improves considering only Pacific samples (n = 13, R = 0.69, *p* = 0.0058, Fig. 6c, in bold). We then performed a multiple regression analysis only with Pacific samples (n = 11) to understand the relative importance of C1, AOU, and bacterial biomass on *G. minor* biomass. We tested separately C1 and AOU because they were strongly correlated. Considering bacterial biomass and C1, the model explained 83% of the variability of *G. minor* biomass, with both parameters being significant predictors (*p* of C1 = 0.0028, *t*-stat =  − 4.25; *p* of bacteria biomass = 0.0378, *t*-stat = 2.48); considering a significance threshold of 0.05, after the Bonferroni correction the bacterial biomass failed to pass the significance test whereas C1 remained significant. Using AOU and bacterial biomass, the model explained 87% of the variability of *G. minor* biomass, being AOU the only significant predictor (*t*-stat =  − 5.47, *p* = 0.0004).

### Abiotic parameters

Abiotic parameters (temperature, salinity, conductivity, and oxygen) did not have a significant correlation with *G. minor* biomass both for the global bathypelagic ocean and considering only Pacific samples. Higher values of biomass are within a narrow range of values both for salinity (around 34.7 psu) and oxygen (3.23–3.58 mL L^−1^), which is expected considering that they all belong to the same water mass.

## Discussion

The presence of a clear hotspot for *G. minor* in the bathypelagic region of the Equatorial Pacific has been demonstrated by consensus using two different sequencing datasets and a species-specific TSA-FISH probe. Since the ASV retrieved from the sequencing analysis was 100% similar to a cultured strain of *G. minor,* it was possible to test the probe prior to its application to environmental samples. The TSA-FISH technique allowed us to visualize the morphotype (life-stage) of the targeted fungi, showing that the majority of *G. minor* cells were rounded, with a single nucleus and unstained with WGA (Fig. 2). Larger elongated cells were also observed but at much lower abundance.

*Gjaerumia minor* belongs to the class *ustilaginomycetes,* which are usually dimorphic, producing a saprobic haploid yeast phase and a parasitic dikaryotic phase^48^, so particular attention was given to the identification of the proper life stage of the rounded cells. If they corresponded to a resting stage (teliospore) it would mean that *G. minor* is not thriving in the bathypelagic environment, whereas a yeast phase opens the possibility that this fungus is alive and active in those waters. Three hints suggested us to exclude the teliospore hypothesis: (i) their size is too small; although teliospores from the sister species *Gjaerumia ossifragi* (Bauer 2005, Figs. 5–14)^49^ show a rounded shape, they are ten times larger than the ones retrieved here and, in proportion with the size of the hyphae, rounded cells seem too small to be teliospores, (ii) they are not dikaryotic as a teliospore is expected to be; we never observed two nuclei in DAPI stained samples, (iii) last and most relevant, rounded cells are not stained by WGA, a specific stain for chitin. Teliospores, in turn, are expected to have a very thick chitin wall (Fig. 2g–i). The lack of WGA staining is probably due to the fact that yeast merging and budding are favored by a thinner chitin wall. Another possibility is that the rounded morphotype is a reproductive spore, such as basidiospore (sexual) or basidioconidia (asexual). Although both spores are expected to be fusiform^49,50^ it is possible that the pressure of the bathypelagic environment could force a more globose shape^41^.

*Gjaerumia minor* is known to have a strong plasticity being found as plant parasites^18,19^ and human pathogens^20–22^. In the frame of Malaspina 2010 cruise, it is often present also in surface waters (51 of 136 stations), although in a lower percentage. This is comparable with *Tara ocean* results, an expedition that sailed roughly at the same time as *Malapina* and that investigated the microbial diversity of the photic zone using the V9 region of the 18S rDNA^51^. In that study, an OTU belonging to the *Gjaerumia* genus (*Tilletiopsis albescens*) was found only 24 times in surface waters. It is clear that *G. minor* is not an endemic species of the bathypelagic environment but is transported there from land through the surface global ocean. For prokaryotic communities, based on samples from the same cruise, it has been reported a direct connectivity through fast-sinking particles between surface and bathypelagic layers^52,53^. We hypothesized a similar mechanism of transport for *G. minor*, as previously suggested by Bochadansky et al.^9^. This idea is also reinforced by the fact that the osmotrophic food acquisition in fungi, based on the secretion of extracellular enzymes, better suits a particle-attached lifestyle^6^. The particle-attached lifestyle was further supported by the fact that G. minor showed increased abundance as the size fraction of DNA samples analysed in Pacific bathypelagic waters increased, with maximum values in the largest size fraction considered, 5–20 µm (Fig. S2).

These particles could represent sinking *G. minor* hosts (phytoplankton) or fragments of them (animals or macroalgae). Species taxonomically close to *G. minor* have been found associated with sea-animals^10,11^, macroalgae^12^ and even dinoflagellates^54,55^. Related to this, data of the Malaspina cruise^56^ reported high concentrations of large phytoplankton in the deep sea, represented by living fast-sinking cells (81.5% were diatoms, followed by dinoflagellates) with the highest abundance values found in the Equatorial Pacific region, opening the hypothesis of undiscovered microbial interactions. Interestingly, the sequencing analysis show an absence of *G. minor* at the surface in a large area of the equatorial Atlantic Ocean, from 14° N to 24° S (stations 17–26). So, the lower presence of this species in Atlantic bathypelagic waters could be explained by its absence at the surface, as the snowfall of particles at the equator is similar between the two oceans^57^.

Despite several hints pointing to the fact that fungi arrive to the deep ocean attached to particles, we have been able to observe this phenomenon only once (Fig. 2e,f, which shows a large particle colonized by rounded cells), while most observed rounded cells appear to be free-living. There are possible methodological reasons to explain the lack of particles observed in our TSA-FISH samples (low volume, high filtering pressure). Nevertheless, even in an environment full of particles, yeasts are expected to search for new resources once the ones of their transport particle are exhausted. We propose that, although sample manipulation could separate some cells from particles, part of the bathypelagic community live temporarily in a free-living state as evidenced by past results of flow cytometry^16^, sequencing of the 0.2–0.8 µm size fraction (often used as the free-living size fraction for bacteria, supplementary Table S1) and the TSA-FISH itself.

The parameter that seems to better explain *G. minor* distribution in the Pacific area is AOU (Figs. 5 and 6a). Considering only Pacific samples, *G. minor* was more abundant in the less-aged waters. It is possible that in these waters, *G. minor* finds a mix of conditions that we failed to singularly detect, that allow its prosperity. As AOU and the component C1 of FDOM (proxy for recalcitrant carbon) are strongly correlated, it is difficult to understand their respective contribution to *G. minor* distribution. Our analysis showed a significant inverse correlation between *G. minor* biomass and the C1 peak in Pacific waters (Fig. 6b), which opens the possibility that *G. minor* contribute to the consumption of recalcitrant carbon. Interestingly, the relation between C1 and bacterial biomass is not significant considering the same set of samples. It has been suggested that fungi are often better than bacteria at breaking down recalcitrant organic material^58^. Although the biomass of *G. minor* is much lower than the biomass of bacteria, it is still possible that they both contribute to the degradation in a mutualistic rather than antagonistic way^9,59^. Perhaps the interaction of *G. minor* with specific prokaryotic clades could enhance the utilization of C1 in a way reflected more by the fungal biomass (as proxy of this interaction) rather than the entire bacteria biomass.

For the complete dataset, the biomass of free-living bacteria is not a good predictor of *G. minor* distribution in our multiple regression analysis. However, considering only the equatorial Pacific samples, bacterial biomass is well correlated with *G. minor* yeast cells (Fig. 6c). Differently from Bochadansky et al.^9^, who found similar values for the two groups, here fungal biomass is 3 orders of magnitude lower than the bacterial one. This prokaryotic free-living pool represents the endemic part of the community and, in contrast with the particle attached assemblage, does not correlate with surface biotic variables but with bathypelagic environmental conditions^53^. This leads us to propose that the good correlation between the biomass of *G. minor* and free-living bacteria in Pacific samples is due either to a parallel response to the bathypelagic conditions and resources or to the utilization by fungi of carbon processed by bacteria. In fact, both cases point to an active free-living fungal community. In culture conditions, *G. minor* does not grow without some vitamins^50^, and in particular it needs thiamine to thrive. It is possible that the prokaryotic community could be the source of these vitamins and a reliable scenario is that vitamin availability improves the conditions for *G. minor,* which could grow and reproduce in marine deep waters.

The idea that once passively transported to the bathypelagic layer of the Pacific, *G. minor* is capable of establishing an actively thriving population is supported by several hints. First, we know from experimental evidence in *Saccharomyces cerevisiae* that fungi can alter their membrane composition to tolerate high hydrostatic pressures^60^, which could make possible a rapid colonization of deep-sea habitats by surface strains^61^. Second, considering the entire dataset, not only Basidiomycota but also Ascomycota tags peak in the aged waters. Moreover, the Ascomycota peak is spatially displaced forward compared to the Basidiomycota peak, pointing to different target substrates and reinforcing the idea of an active fungal deep community. Third, a recent study exploring the potential utilization of different carbon sources by the microbial community using Biolog GN2 plates found a major utilization of polymers (Dextrin, a-Cyclodextrin, Glycogen, Tween-40 and Tween-80, Fig. S3 from Sala et al.^62^) in *G.minor* dominated waters, suggesting the capability of these communities to process complex DOM. These compounds are known to be processed by several species of fungi^63–66^, suggesting again the presence of an active fungal community in deep oceanic waters.

The presence of the cultured plant-parasite *G. minor* has been highlighted in bathypelagic waters, especially in the Equatorial Pacific where its biomass peaks, both by species-specific TSA-FISH probe and by high-throughput sequencing. Our data suggests that in these waters *G. minor* is present as an active population, probably surviving thanks to some interaction with the bacterial community. Our results show, in accordance to previous works^53,67^, that the bathypelagic ocean is not an isolated environment but it is in continuous connection with surface waters and with the land. In this regard, the fungus *G. minor* shows an enormous potential to be an important active component of the global carbon cycle, pointing at the same time to the importance and necessity of more studies both on the bathypelagic ocean and on its fungal diversity.

### Supplementary Information

Supplementary Legends.

Supplementary Table S1.

Supplementary Figure S1.

Supplementary Figure S2.

## Data Availability

The sequencing datasets analysed in this work all belong to the Malaspina expedition and are already published and publicly available at the European Nucleotide Archive (ENA). Amplicon sequences from 454-pyrosequencing of the deep ocean are available at www.ebi.ac.uk/ena/data/view/PRJEB9943; Illumina sequences from vertical profiles at www.ebi.ac.uk/ena/data/view/PRJEB23771; Illumina sequences from surface samples at www.ebi.ac.uk/ena/PRJEB23913 and Illumina sequences from the deep ocean at www.ebi.ac.uk/ena/data/view/PRJEB45014.
