# Supplementary material for: A fungi hotspot deep in the ocean: explaining the presence of Gjaerumia minor in equatorial Pacific bathypelagic waters
Source: Sci Rep. 2024 May 8;14:10601. doi: 10.1038/s41598-024-61422-7 (PMC11079054; doi:10.1038/s41598-024-61422-7)
Supplement: Supplementary file 1 — Supplementary Legends. [file 41598_2024_61422_MOESM1_ESM.pdf]

Figure S1: Map of Malapina stations used by this work. Stations where a sequencing analysis of the vertical profile was performed, are marked by a number. Stations analyzed with TSA-FISH are circled in purple, the maximum value of cell abundance is indicated in red. This map was done with Ocean Data View, version 5.4 (https://odv.awi.de/).

Figure S2: Boxplot of Illumina tags belonging to *G. minor* from a size fraction analysis, the numbers on the horizontal axis are expressed in µm. This picture is built from a metabarcoding 18S rDNA dataset, not yet public, prepared from the samples taken and analyzed in Mestre et al. 2018[51].
