# Supplementary figures and images for: A fungi hotspot deep in the ocean: explaining the presence of Gjaerumia minor in equatorial Pacific bathypelagic waters
Source: Sci Rep. 2024 May 8;14:10601. doi: 10.1038/s41598-024-61422-7 (PMC11079054; doi:10.1038/s41598-024-61422-7)

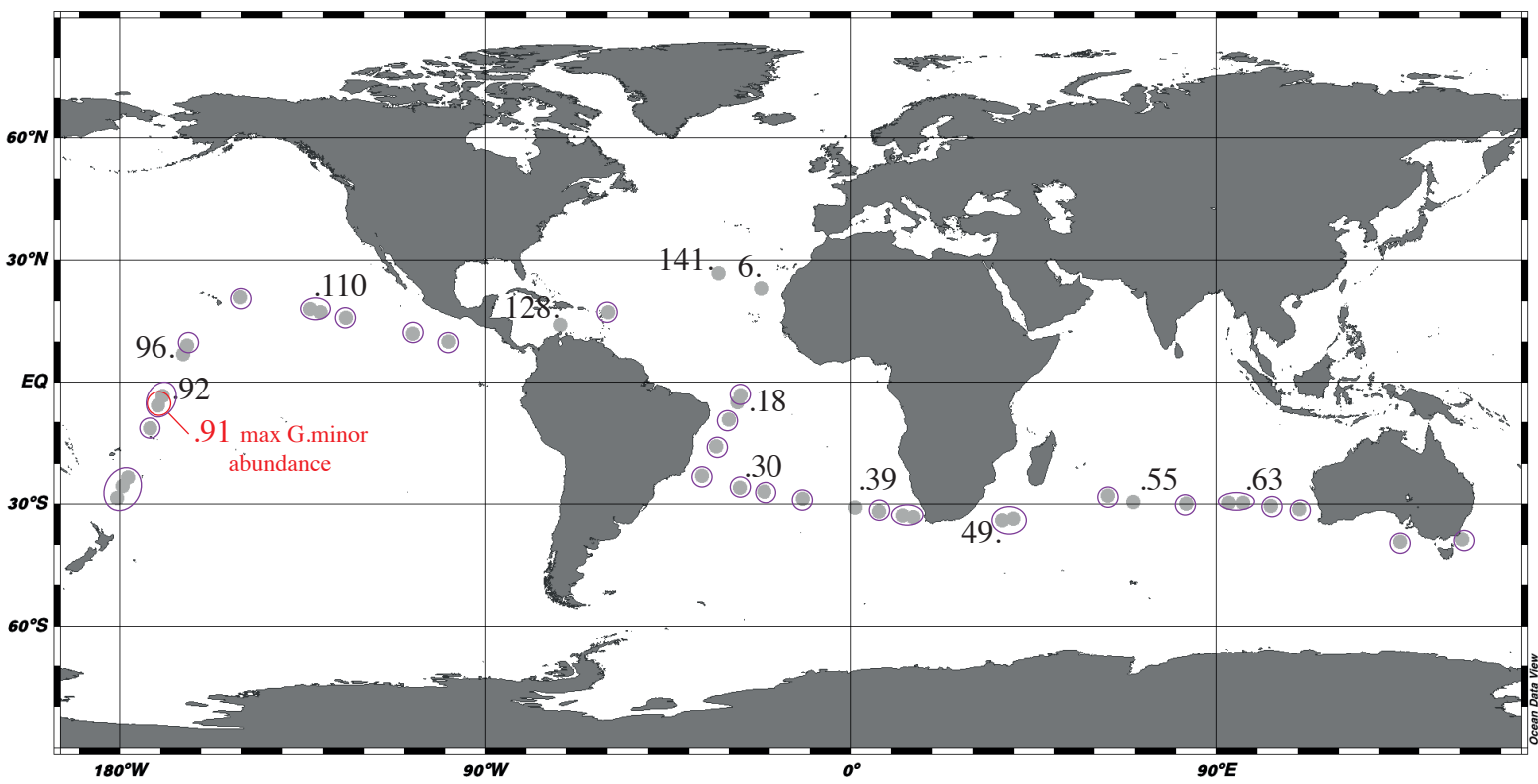

Supplement: Supplementary file 3 — Supplementary Figure S1. [file 41598_2024_61422_MOESM3_ESM.pdf]

## Size Fraction

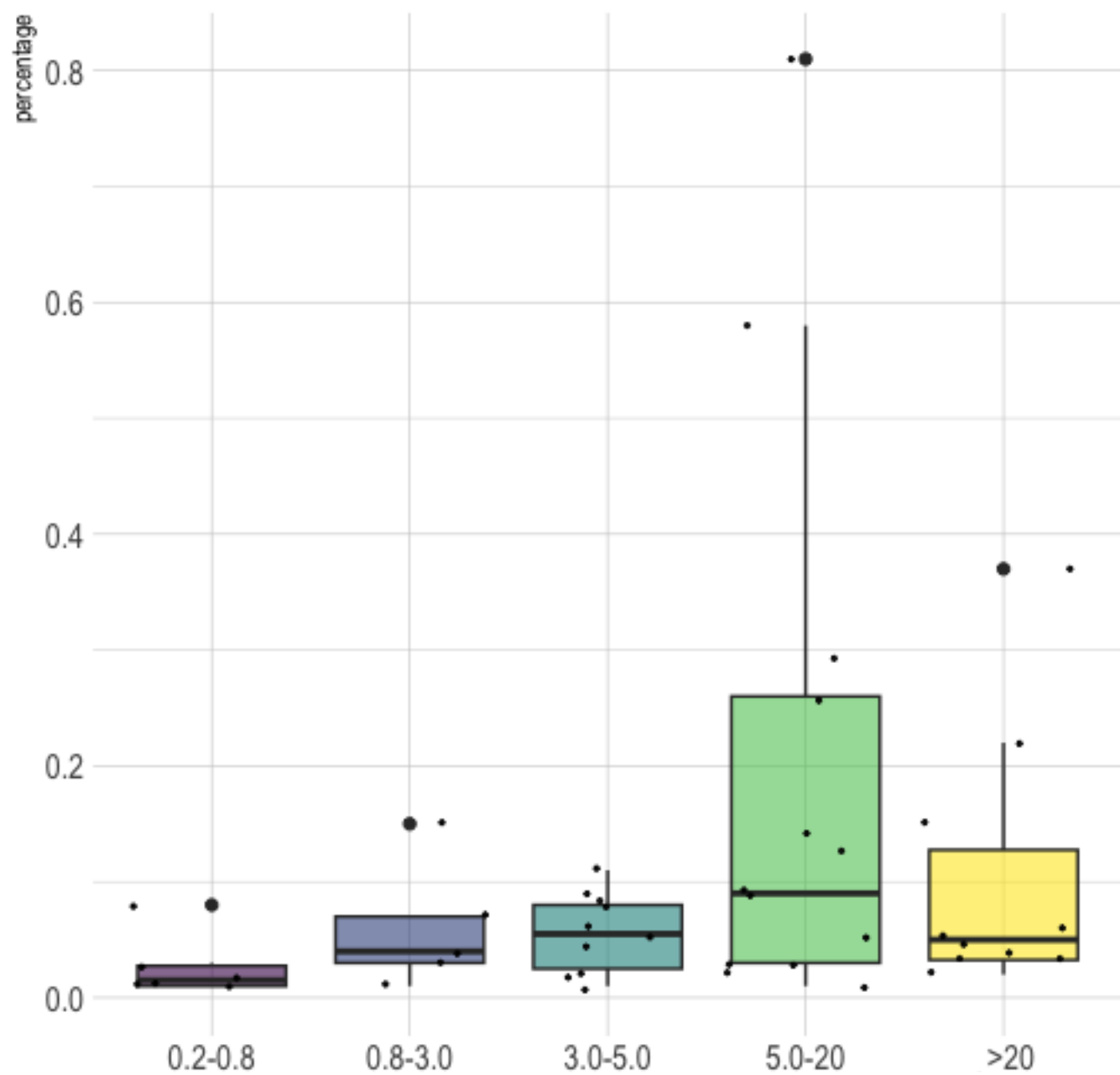

Supplement: Supplementary file 4 — Supplementary Figure S2. [file 41598_2024_61422_MOESM4_ESM.pdf]
